# Endoscopic Microvascular Decompression for Vagoglossopharyngeal Neuralgia

**DOI:** 10.7759/cureus.12353

**Published:** 2020-12-29

**Authors:** Rachel Blue, Michael Spadola, Michael McAree, Svetlana Kvint, John Y.K. Lee

**Affiliations:** 1 Department of Neurosurgery, University of Pennsylvania, Philadelphia, USA; 2 Department of Neurosurgery, Rowan School of Osteopathic Medicine, Philadelphia, USA

**Keywords:** vagoglossopharyngeal neuralgia, glossopharyngeal neuralgia, microvascular decompression, endoscope, syncope

## Abstract

Glossopharyngeal neuralgia (GN) is a nerve compression syndrome that presents with episodes of unilateral sharp, stabbing pain in the distribution of the ninth cranial nerve. This syndrome may present with cardiac and autonomic manifestations - a condition termed vagoglossopharyngeal neuralgia (VGPN). Most cases of VGPN arise from neurovascular insult at the cerebellopontine angle. Conservative treatment for VGPN includes antiepileptic medications. Surgical treatments include trigeminal tractotomy-nucleotomy, Gamma Knife® stereotactic radiosurgery, radiofrequency thermocoagulation, rhizotomy, and, as shown in this paper, endoscopic microvascular decompression (E-MVD). In this article, we present two cases. Case 1 demonstrates a 53-year-old male with right-sided GN symptoms that began to experience syncopal episodes 10-years after the initial presentation. Case 2 presents a 61-year-old female with a history of Ehlers-Danlos syndrome, and the malignant vasovagal syndrome that became associated with painful, shooting left anterior neck spasms consistent with GN. Both patients underwent E-MVD, leading to complete relief of neuralgia and cardiac symptoms. Our outcomes support previously published reports of successful treatment of VGPN using microvascular decompression (MVD) and describe a purely endoscopic surgical technique. MVD is the preferred treatment option for VGPN with evident neurovascular insult.

## Introduction

Glossopharyngeal neuralgia (GN) is a nerve compression syndrome with an incidence of 0.8/100,000 and typically presents with episodes of unilateral sharp, stabbing pain in the distribution of the ninth cranial nerve, including the throat, jaw, ear, and tongue [1]. Rarely, sensory rootlets of the vagus nerve may be affected, leading to associated syncope, arrhythmia, or additional cardiac and autonomic manifestations; this condition is termed vagoglossopharyngeal neuralgia (VGPN) [1, 2]. Conservative treatment for VGPN includes the use of antiepileptic medications. Surgical treatment options include microvascular decompression (MVD). Historically, microscopic MVD has been the approach for resolving neurovascular insults at the cerebellopontine angle (CPA). However, a promising role for endoscopic MVD (E-MVD) exists, as it may identify insults missed by a purely microscopic approach and offers a minimally invasive approach with improved visualization, particularly around anatomic corners and where lighting is poor. While an endoscopic approach for MVD has been reported in treating other cranial nerve compression syndromes, including trigeminal neuralgia, hemifacial spasm, and GN, VGPN is less commonly reported [3]. Here we present the successful treatment of two patients with symptomatic VGPN treated with purely endoscopic MVD [4].

## Case presentation

Case 1

Case 1 demonstrates a 53-year-old male with right-sided GN symptoms that began to experience syncopal episodes 10-years after initial presentation. The patient underwent extensive medical and cardiac workup prior to surgical consideration. He was diagnosed with supraventricular tachycardia and underwent cardiac ablation, but continued to experience syncopal episodes despite the resolution of his tachyarrhythmia. Preoperative MRI was unrevealing for neurovascular compression. 

**Figure 1:**
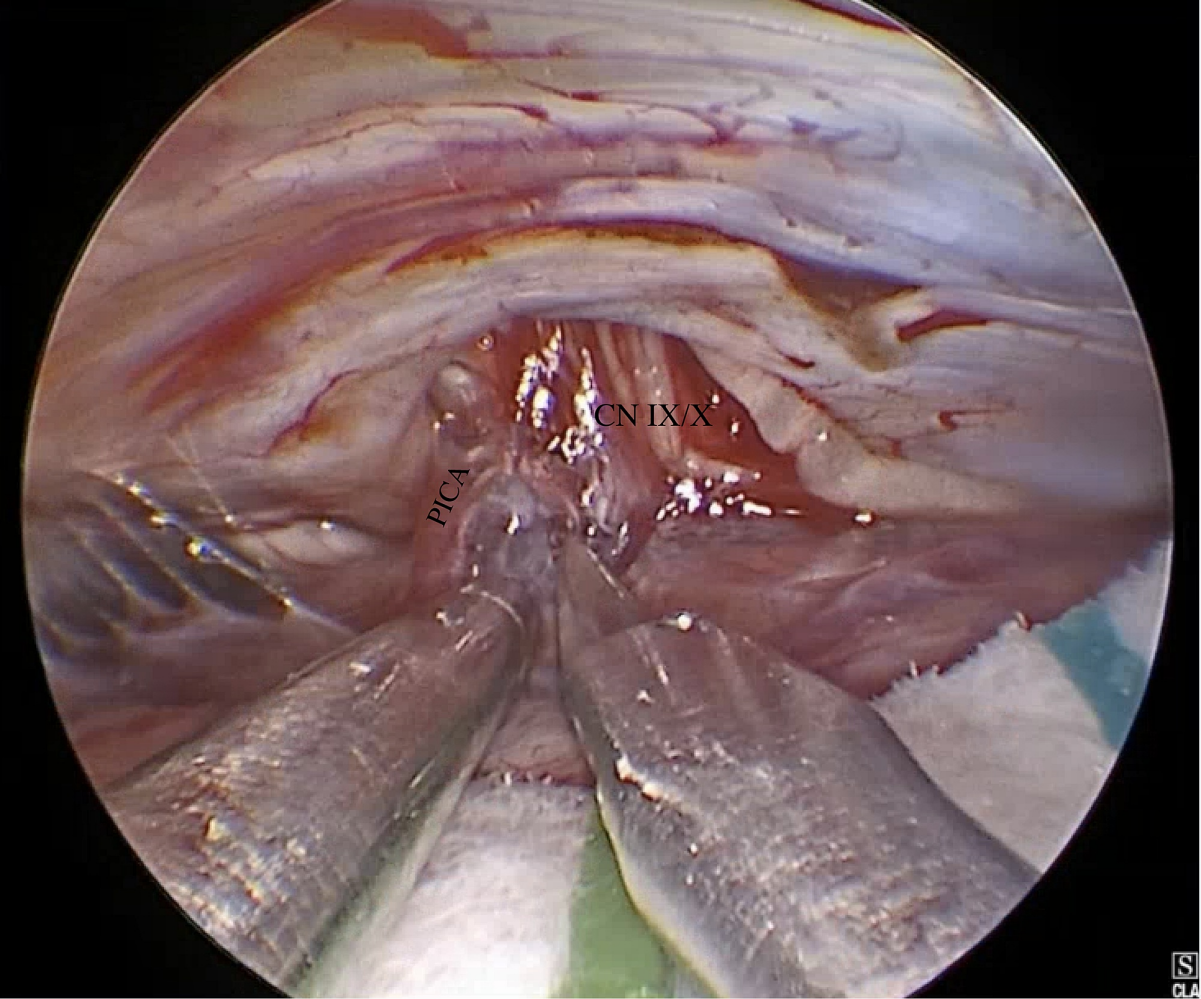
Compression of the glossopharyngeal nerve by posterior inferior cerebellar artery CN - cranial nerve; PICA - posterior inferior cerebellar artery

Ultimately, he underwent a right retrosigmoid craniotomy for E-MVD. During the procedure, it was noted (see Figure 1) that the glossopharyngeal nerve was under compression by the posterior inferior cerebellar artery (PICA), which was decompressed using polytetrafluoroethylene (PTFE). This patient had an uncomplicated postoperative course and was seen at follow-up without a return of neuralgia or syncopal episodes. At 18 month follow-up, he had no recurrence of cardiac syncope.

Case 2

**Figure 2:**
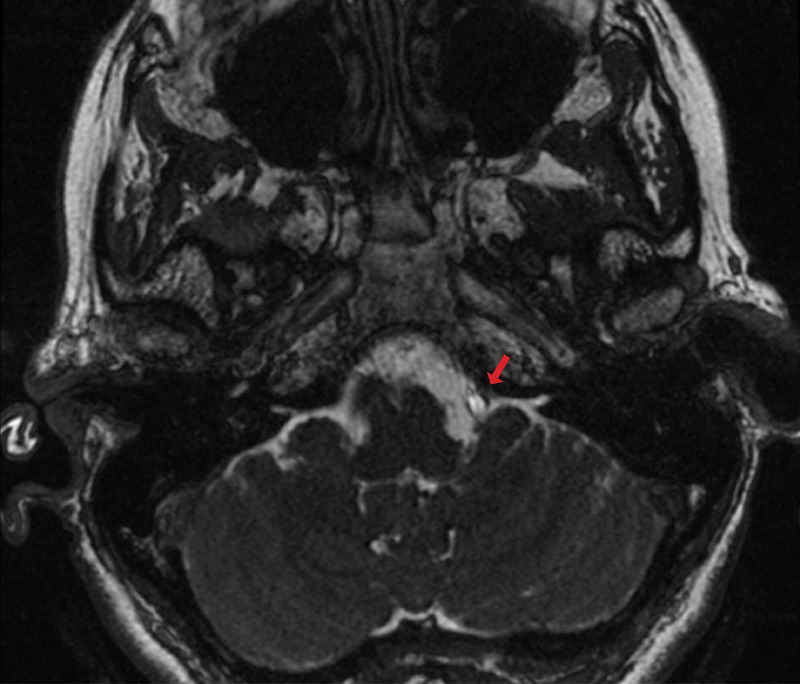
Preoperative MRI demonstrating a left dominant vertebral artery (arrow) causing compression on cranial nerves IX and X

Case 2 demonstrates a 61-year-old female with a history of Ehlers-Danlos syndrome, and a malignant vasovagal syndrome that became associated with painful, shooting left anterior neck spasms consistent with GN. This patient underwent extensive medical and cardiac workup prior to surgical consideration. Additionally, she underwent bilateral styloidectomy for concerns of Eagle syndrome but continued to have both neuralgia and cardiac symptoms. MRI demonstrated a dominant left vertebral artery causing vascular compression on the lower cranial nerves (Figure 2).

**Figure 3:**
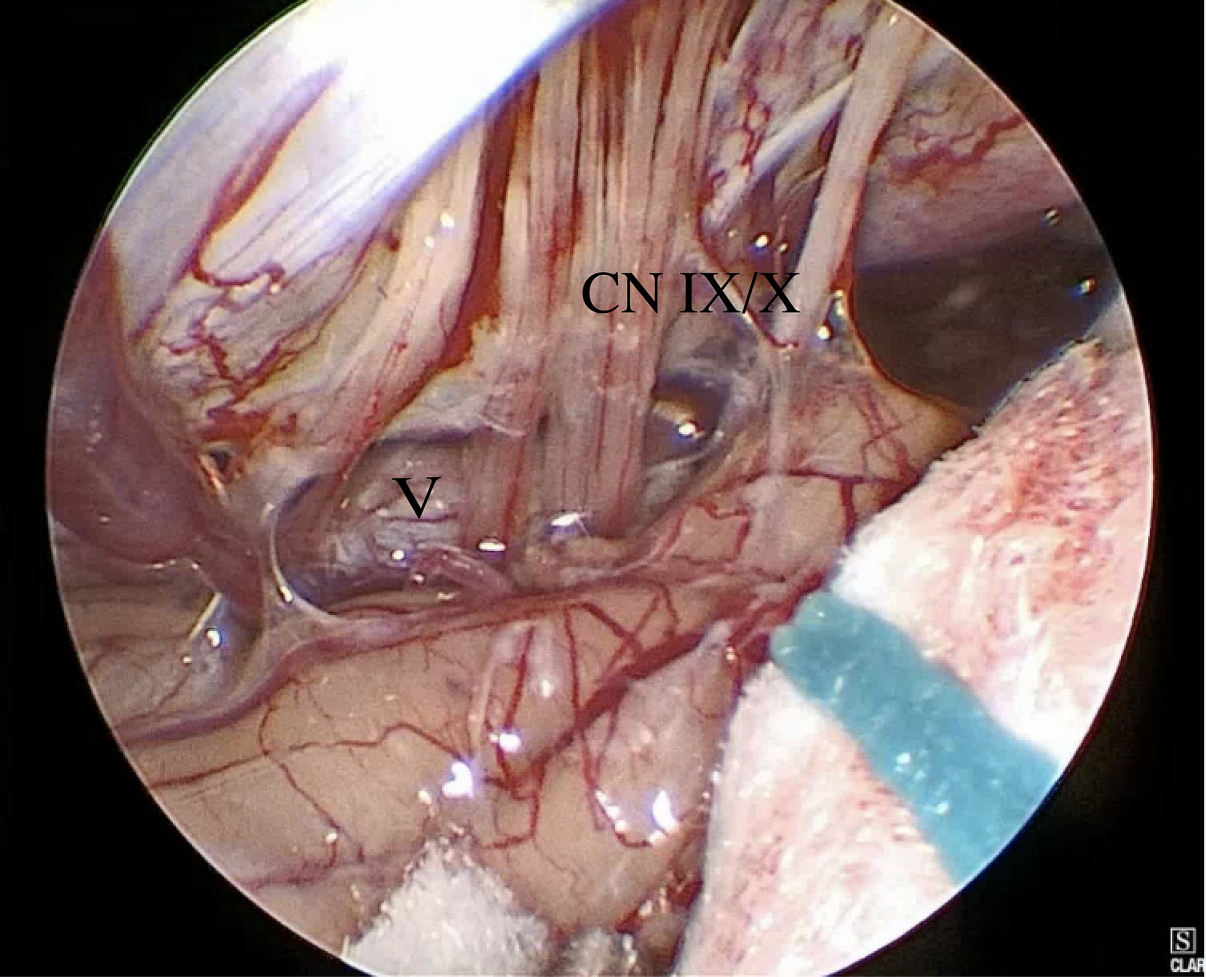
Compression of the glossopharyngeal nerve by the vertebral artery CN - cranial nerve; V - vertebral artery

Ultimately, she underwent retrosigmoid craniotomy for E-MVD (see Video 1), which revealed glossopharyngeal nerve compression by the vertebral artery (Figure 3), which was decompressed using polytetrafluoroethylene (PTFE). She had an uncomplicated postoperative course and was seen at follow-up without a return of neuralgia or syncopal episodes. At a nine-month follow-up, she had no recurrence of cardiac symptoms.

**Video 1:** Endoscopic microvascular decompression for vagoglossopharyngeal neuralgia

## Discussion

Surgical technique

All operations are performed under general anesthesia. The head is secured with the Mayfield clamp, and the patient is placed in a lateral position. Neuronavigation and lumbar drainage are not necessary. Anatomical landmarks are identified for the linear skin incision and after muscle dissection, including the tip of the mastoid process and the digastric notch. A single burr hole approximately 1.5-2 cm in diameter is made inferior to the sigmoid transverse sinus junction. A C-shaped dural incision is created and flapped towards the sigmoid sinus and secured with a single stitch.

Following the dural opening, a cottonoid patty is inserted for gentle retraction on the cerebellum, and careful arachnoid dissection allows to release of the cerebrospinal fluid. A 2.7 mm outer diameter offset endoscope (Storz) is fixed into place with the aid of a Mitaka Pneumatic Arm (Mitaka Kohki Co., Ltd, Tokyo, Japan), allowing for bimanual dexterity and enhanced surgical ergonomics throughout the case. Fixed endoscope stabilization prevents unintended injury of surrounding neurovascular structures. As previously described, a strict triangle technique is used, with the endoscope fixed at the apex of the visualization triangle both proximally at the dura and distally at the petrous temporal bone and jugular foramen. At the entrance of the cerebellar convexity dura, the endoscope must be at the apex at "2 o’clock", and the instruments can be passed underneath the endoscope along the cerebellar folia surface at "5 o’clock" and "7 o’clock" [5].

This triangle method allows for sharp dissection at the root entry zone of the glossopharyngeal and vagal nerves and excellent visualization of the neurovascular conflict. Once the arachnoid has been dissected off the nerves, polytetrafluoroethylene (PTFE) is used to decompress the neurovascular conflict. Dynamic retraction is used through the entirety of the case, avoiding the use of fixed brain retractors. Following decompression, the surgical field is filled with saline, and the dura is closed in a watertight fashion.

Pathogenesis

While the pathogenesis of VGPN remains debated, one commonly accepted cause is the neurovascular insult of the ninth and tenth nerve rootlets as they emerge from the brainstem at the root entry zone (REZ) [6]. As the tenth nerve emerges, it enlarges to form a superior ganglion and proceeds inferiorly, communicating with the ninth nerve [7]. Secondary causes include the presence of tumors, mass effect, aneurysms, inflammation, and adhesions of the arachnoid, persistent hypoglossal artery, vagal root neuromas, eagle syndrome, or persistent inflammation of the temporal bone [2, 8-11].

The mechanism of how pain in the glossopharyngeal distribution and vagal symptoms overlap remains unclear. One proposed mechanism is that rootlet adhesions between the ninth and tenth nerves disturb the carotid sinus nerve of Hering, which stimulates the motor vagal nucleus enough to promote syncope [1]. Others postulate that the vascular insult causing pain may stimulate the ninth nerve enough to slow cardiac rhythm and/or increasing vagal nucleus sensitivity.

Treatment options

Conservative treatment of VGPN involves the use of antiepileptic medications, such as carbamazepine, oxcarbamazepine, valproic acid, lamotrigine, and levetiracetam, to control pain [12]. Beta-agonists and anticholinergics, such as isoproterenol and atropine, may be used to control bradycardia and autonomic related symptoms [13]. Refractory cases of VGPN may be treated using a variety of surgical techniques [14].

The trigeminal tractotomy-nucleotomy procedure involves the destruction of the trigeminal tractus of the medulla at the level of the inferior olive [2]. While it has been successful in providing relief, it should be reserved for circumstances of malignant pain or neuralgias due to tumor mass effect because of the risks associated with the procedure. Gamma Knife stereotactic radiosurgery (GKS) targeting the REZ, cistern segment, pars nervosa of the jugular foramen, or medial cisternal segments of the ninth nerve can also be performed [15]. While it is less invasive than MVD, it historically involves a higher recurrence rate. For this reason, GKS should only be reserved for circumstances where a surgical approach is contraindicated. Percutaneous radiofrequency thermocoagulation, a technique that carries an increased risk of damaging the jugular vein and the internal carotid artery as well as sensorimotor deficits, is becoming obsolete with the advent of surgical treatment options such as MVD [16].

MVD is the preferred treatment for GN and VGPN with neurovascular insult. This is commonly due to arterial compression along with central myelin of the ninth nerve and rostral fascicles of the tenth nerve. The posterior inferior cerebellar artery (PICA) is a common culprit vessel in GN and VGPN; other cerebellar arteries may be implicated [16]. Vascular decompression is often curative, demonstrating a total relief rate greater than 90% and lower recurrence rates when compared to other surgical interventions [2]. Procedurally, retrosigmoid craniectomy may be performed. Subsequent opening of the arachnoid at the cerebellomedullary cistern allows for exposure of the ninth and tenth nerve at the REZ. However, unlike in decompression of the trigeminal, facial, or abducens nerve, the retrosigmoid approach may result in difficulty visualizing neurovascular compression and the full course of the ninth nerve. Lateral, transcondylar, and extreme lateral approaches to the brainstem can be considered to help improve visualization. The use of a subtonsillar approach has also been reported, as it provides an excellent view of the neurovascular conflict. This is because the entire intracisternal course of the ninth nerve bundle may be exposed, and the jugular bulb and foramen may be successfully avoided.

In our practice, the use of a fully endoscopic approach allows for the continued use of a minimally invasive retrosigmoid approach while achieving improved visualization of the entire course of the ninth nerve compared to the microscopic approach. The use of the endoscope for GN has been described, noting improved visualization of neurovascular conflict compared to microscopic approaches [17-20]. The use of the endoscope in VGPN is not well described, but the approach and benefits are similar to that of GN. The use of the endoscope allows for improved surgical maneuverability, panoramic visualization, and the ability to see around corners with the use of angled lenses.

Another surgical option to consider includes intraoperative rhizotomy of the ninth and tenth nerves. This procedure should only be indicated for patients without a clear neurovascular insult. While it is still a destructive procedure, unlike thermocoagulation, unwanted outcomes such as pharyngeal discomfort, dysphagia, and hoarseness may be mitigated if rhizotomy of tenth nerve fibers can be avoided [14]. In combination treatment, when considering whether or not to section vagal nerve rootlets, the use of electrophysiological monitoring is imperative. If vagal rootlets contain motor innervation, decompression should be permitted, while sectioning should be avoided. If vagal rootlets contain solely sensory innervation based on neuromonitoring, sectioning may be permitted. Overall, MVD alone or in combination with additional surgical approaches have been shown to provide relief of symptoms in patients with GN and VGPN, and our experience has shown the use of an endoscope allows improved visualization of neurovascular conflict while maintaining minimal surgical exposure.

## Conclusions

We presented two cases demonstrating safe and successful management of VGPN with E-MVD after the failure of medical therapy. These outcomes support previously published case reports of successful treatment of VGPN using MVD. The use of the endoscope allows panoramic visualization of neurovascular conflict and improved surgical maneuverability compared to that of the microscopic approach.
